# Juveniler Parkinson und Mikrodeletionssyndrom 22q11.2

**DOI:** 10.1007/s00115-022-01426-8

**Published:** 2023-01-03

**Authors:** Carla Palleis, Annika Eißner, Stefanie Förderreuther, Kai Bötzel, Johannes Levin, Adrian Danek

**Affiliations:** 1 Neurologische Klinik und Poliklinik, LMU Klinikum, Campus Großhadern, Ludwig-Maximilians-Universität München, Marchioninistr. 15, 81377 München, Deutschland; 2 Deutsches Zentrum für Neurodegenerative Erkrankungen (DZNE) Standort München, Feodor-Lynen-Str. 17, 81377 München, Deutschland; 3 Munich Cluster for Systems Neurology (SyNergy), München, Deutschland; 4 Klinik für Neurologie, Helios Klinikum München West, Steinerweg 5, 81241 München, Deutschland

## Hintergrund

Das Mikrodeletionssyndrom 22q11.2 (22q11.2 Mikrodeletion), auch DiGeorge-Syndrom, stellt mit einer Frequenz von 1:3000–6000 Geburten die häufigste Mikrodeletion beim Menschen dar [4, 8]. Es liegt zumeist eine Spontanmutation mit heterozygoter Mikrodeletion im Bereich 22q11.2 vor. In über 90 % der Fälle sind „low copy repeats“ im Bereich LCR22A–LCR22H auf Chromosom 22q11.2 betroffen [9]. Die klassische Manifestation des vielseitigen Phänotyps beinhaltet unter anderem Anomalien des Gaumens (Gaumenspalte, Lippen- und Gaumenspalte, velopharyngeale Insuffizienz), angeborene Herzfehler (konotrunkale Fehlbildungen und Ventrikelseptumdefekte), Hypokalzämie, faziale Dysmorphien, Thymushypoplasie, epileptische Anfälle, Entwicklungsverzögerung und psychiatrische Störungen wie Schizophrenie [4, 8]. Die 22q11.2-Mikrodeletion wird zudem als potenzieller Risikofaktor für die Entstehung eines juvenilen Parkinson-Syndroms („Early-onset-Parkinson-Erkrankung“; EOPD) mit Erkrankungsalter vor dem 50. Lebensjahr beschrieben [1–3, 5, 8]. Wir berichten über einen Patienten, bei dem eine äußerst frühe Parkinson-Manifestation mit psychiatrischen Komorbiditäten vorlag.

## Fallbeschreibung

Bei Erstvorstellung im Alter von 25 Jahren berichtete der ausgebildete Hotelfachmann über eine seit einem Jahr bestehende Gangstörung mit Starthemmung sowie schwerfälligen und verlangsamten Bewegungen der Extremitäten, zudem über ein Zittern der rechten Hand. Er gab einen Alkoholkonsum von zwei Bier pro Tag an. Es bestand keine Anamnese von Dopaminantagonisten. In der klinisch-neurologischen Untersuchung zeigten sich eine rechtsbetonte Bradykinese mit intermittierendem Ruhe- und Haltetremor, ferner ein distal und rechts betonter Rigor beider Arme, Freezing sowie eine Dysarthrophonie.

Im Alter von 15 Jahren war eine heterozygote De-novo-22q11.2-Deletion (Karyotyp: 46,XY.ish 22q11.2 [TUPLE1/D22S553/D22S609/D22S942/D22S75-]) nachgewiesen worden. Als Vorerkrankungen waren ein perimembranöser Ventrikelseptumdefekt, Hypoparathyreoidismus mit Hypokalzämie und rezidivierende bronchopulmonale Infekte seit dem 4. Lebensjahr bekannt. Geburt und frühkindliche Entwicklung seien normal gewesen. Es bestand keine auffällige faziale Dysmorphie, allerdings ähneln Schädelform und Gesichtszüge unseres Patienten durchaus denjenigen von publizierten Fällen mit 22q11.2-Mikrodeletion.

## Erweiterte Diagnostik

Logopädisch zeigte sich im Initialbefund eine Dysarthrie mit leichter Artikulationsunschärfe sowie beschleunigtem Sprechtempo. In der Tremoranalyse hatte der rechtsseitige Ruhetremor eine Frequenz von 6,9 Hertz. Der initiale neuropsychologische Befund war unauffällig. Nach 23 Monaten zeigte sich eine depressive Symptomatik mit dadurch erklärbarer erhöhter Anzahl von Fehlern bei selektiven Aufmerksamkeitsleistungen bei sonst unauffälligen Befunden in den Domänen Gedächtnis, exekutive Funktionen und Wahrnehmung/Visuokonstruktion.

Laborchemisch ergaben sich keine Auffälligkeiten, insbesondere nicht im Hinblick auf Morbus Wilson oder auf bekannte Genmutationen, die ein Parkinson-Syndrom verursachen (*ATP13A2*, *ATP1A3, CHCHD2, FBXO7, GCH1, LRRK2, MAPT, PARK7, PINK1, PLA2G6, PRKN, SNCA*).

**Figure 1:**
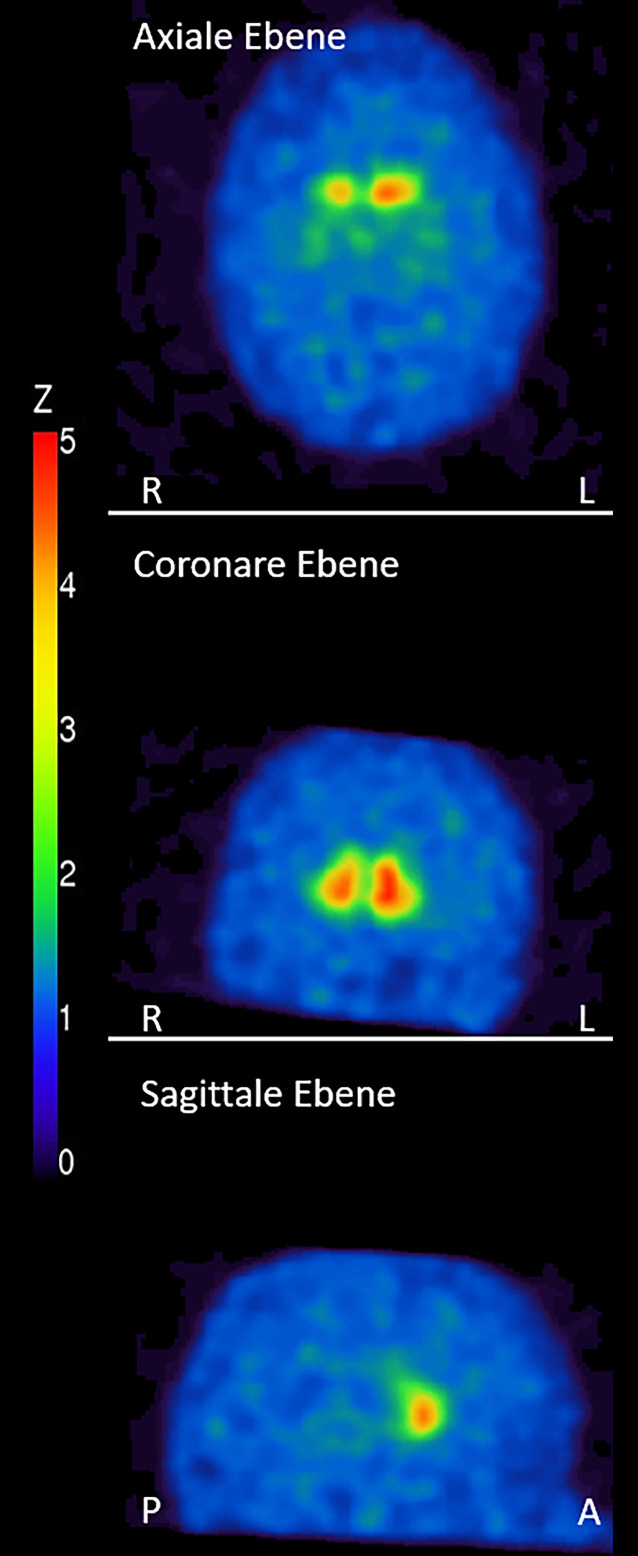

Die MRT des Kopfes zeigte neben einer temporopolaren Arachnoidalzyste links keine Auffälligkeiten, insbesondere keine Kalzifikationen oder Veränderungen der Basalganglien. Im FP-CIT-SPECT mit 181MBq ^123^I lag beidseits eine hochgradig reduzierte Aktivitätsakkumulation im Striatum vor. Der Putamen/Nucleus-caudatus-Quotient war beidseits reduziert (Abb. 1). Eine Gehirn-PET mit 120MBq ^18^F‑FDG ergab keinen Hinweis auf einen kortikalen Glukosehypometabolismus.

## Verlauf und Therapie

Bei hypokinetisch-rigidem Syndrom wurde zunächst Levodopa (150 mg/Tag) eingesetzt. Bei initial gutem Ansprechen wurden wegen des jungen Erkrankungsalters Therapieversuche mit Dopaminagonisten unternommen, mussten aber aufgrund einer beginnenden Impulskontrollstörung (Spielsucht und Alkoholkonsum) beendet werden. Unter Catechol-O-Methyltransferase-Hemmern kam es zu Diarrhöen. Bei im Verlauf von 3 Jahren nach Erstmanifestation rasch progredienten Parkinson-Symptomen sowie Wirkungsfluktuationen wurden zusätzlich zu Levodopa (800 mg/Tag) Amantadin (200 mg/Tag) und Rasagilin (1 mg/Tag) eindosiert.

Im Rahmen der Dosissteigerungen von Levodopa entwickelte der Patient Levodopa-induzierte Dyskinesien. Verhaltensauffälligkeiten unter dopaminerger Therapie sowie erhöhter Alkoholkonsum machten eine psychiatrische Mitbetreuung erforderlich. Aufgrund der konservativ nicht beherrschbaren Wirkungsfluktuationen war die Indikation zur tiefen Hirnstimulation im Alter von 27 Jahren gegeben. Intraoperativ konnte aber mit Mikroelektroden das typische Entladungsmuster des Nucleus subthalamicus weder links noch rechts nachgewiesen werden. Eine intraoperative Stimulation erbrachte keine Besserung von Rigor/Tremor, sodass keine Elektroden implantiert wurden. Der Patient wünschte keine weitere Operation und wurde schließlich mit Levodopa (1000 mg), Amantadin (200 mg) und Rasagilin (1 mg) als Dauermedikation in die ambulante Weiterversorgung entlassen. Bei ambulanter Vorstellung nach einem Jahr war der hypokinetisch-rigide sowie neuropsychologische Befund unter der Entlassungsmedikation stabil. Seit dem 28. Lebensjahr ist es zu keiner erneuten Vorstellung des Patienten gekommen.

## Interpretation

Das Mikrodeletionssyndrom 22q11.2 (22q11.2-Mikrodeletion) wird bei 0,4 % aller Patienten mit juveniler Parkinson-Erkrankung (EOPD) gefunden [2, 5, 8]. Auch wenn es sich bei der 22q11.2-Mikrodeletion um das häufigste beim Menschen bekannte Deletionssyndrom handelt [8], gibt es bislang wenig Anlaufstellen zur Verbesserung der Versorgung von erwachsenen Betroffenen. Maßgeblich beteiligt an der Erforschung und Versorgung erwachsener Patienten ist das interdisziplinäre Referenzzentrum The Dalglish Family 22q Clinic in Toronto, Kanada (https://www.22q.ca/). In einer dort federführend untersuchten, bislang international größten Kohorte von 159 Patienten konnte ein signifikant erhöhtes Auftreten von Parkinson-Erkrankungen (standardisierte Morbiditätsratio: 69,7) gezeigt werden [5]. Es zeigte sich ein deutlicher Effekt des Geschlechts für EOPD mit 72 % männlichen Betroffenen [3]. Bei 84,2 % der Patienten lag dabei wie bei unserem Patienten eine De-novo-Deletion mit unauffälliger Familienanamnese für Parkinson-Erkrankungen vor. Das durchschnittliche Erkrankungsalter lag bei 39,5 Jahren und meist stellten sich, wie im aktuellen Fall, Patienten anfänglich mit einseitigen motorischen Symptomen vor, die gut auf dopaminerge Medikation ansprachen [3]. In einer italienischen Kohorte von 56 Patienten wurden Parkinson-Symptome sogar bei 15 neuroleptikanaiven Patienten (27 % der Gesamtkohorte) mit einem mittleren Alter von 30 Jahren beschrieben [7]. Aufgrund der meist kardial bedingten unterdurchschnittlichen Lebenserwartung von 46,4 Jahren [10] ist davon auszugehen, dass einige Betroffene nicht alt genug werden, um Parkinson-Symptome zu entwickeln. Zudem werden Parkinson-Syndrome bei Patienten mit neuroleptischer Vortherapie später diagnostiziert als bei neuroleptikanaiven Patienten [3].

Es ist bislang nicht klar, welche Genveränderungen durch die Mikrodeletion auf Chromosom 22q11.2 für die Parkinson-Symptomatik ursächlich sind. Als mögliches Risikogen konnte z. B. Catechol-O-methyltransferase identifiziert werden [2]. Jedoch geht man insgesamt in einer ersten Gesamtgenomsequenzanalyse bei 22q11.2-Mikrodeletion davon aus, dass die Akkumulation von Varianten in Genomsequenzen einen schwellensenkenden Effekt zur Entwicklung eines EOPD im Sinne eines „Multi-Hits“ von Parkinson-relevanten Signalwegen hat [6]. Die für Parkinson-Erkrankungen bereits bekannten genetischen Veränderungen lagen dabei in der Genomsequenzanalyse von Patienten mit 22q11.2-Mikrodeletion nicht vor.

In Bezug auf psychiatrische Auffälligkeiten ist bekannt, dass insbesondere eine Deletion im Bereich der LCR22A-B-Region mit einem erhöhten Risiko für eine schizophrene Störung einhergeht [9]. Neuropsychiatrische Symptome bei 22q11.2-Mikrodeletion liegen insgesamt bei bis zu 60 % der Patienten vor [1, 4]. Bereits im frühen Verlauf der Parkinson-Erkrankung treten bei etwa 20 % der Patienten psychotische Symptome, bei nochmals 20 % Depressionen und Angststörungen auf, insgesamt häufiger als beim idiopathischen Parkinson-Syndrom [3]. Dies stellt eine therapeutische Herausforderung dar. Es ist bei 22q11.2-Mikrodeletion schwer zu differenzieren, ob das Auftreten psychotischer Symptome ausschließlich Folge dopaminerger Medikation bei der Parkinson-Erkrankung oder bereits durch die Grunderkrankung bedingt ist [1].

Unser Patient ist der jüngste dokumentierte Patient mit 22q11.2-Mikrodeletion, bei dem sich eine Parkinson-Symptomatik nicht in Folge einer antidopaminergen Therapie entwickelte. Dopaminagonisten waren bei ihm aufgrund einer Impulskontrollstörung nicht mehr einsetzbar. Ein gutes Ansprechen der motorischen Symptome der Parkinson-Erkrankung ist allerdings in der Literatur für dopaminerge Therapie bekannt, wobei rasch progrediente Verläufe wie in unserem Fall beschrieben sind [3, 5, 7]. Da in der Literatur Einzelfälle zur tiefen Hirnstimulation bei 22q11.2-Mikrodeletion vorliegen, erschien uns dies als eine sinnvolle Therapieoption. Immerhin führte die Stimulation im Globus pallidus internus oder Nucleus subthalamicus bei 4 von 5 bislang publizierten Patienten mit 22q11.2-Mikrodeletion zu einem partiellen Ansprechen. Allerdings blieb bei allen Patienten eine dopaminerge Polypharmazie notwendig [3]. Neuroanatomische Besonderheiten, welche die Planung der tiefen Hirnstimulation erschweren, sind bei 22q11.2-Mikrodeletion vorbeschrieben [1]: Cavum septi pellucidi, abnorme kortikale Venen, Polymikrogyrie oder relevante kortikale und subkortikale Atrophien waren in der Bildgebung beim Patienten jedoch nicht zu erkennen. Eine Verkalkung der Basalganglien bei Hypoparathyreoidismus und Hypokalziämie in der Kindheit lag ebenfalls nicht vor. Das Zentrum des Nucleus subthalamicus am Vorderrand des Nucleus ruber befand sich bei unserem Patienten 9 mm lateral der Mittellinie, während der Abstand zur Mittellinie bei den meisten Patienten 12 mm beträgt, sodass dies möglicherweise das intraoperative Fehlen der typischen Entladungsmuster des Nucleus subthalamicus erklärt. Neuerliche Planung und erneute Operation wurden nicht gewünscht. Die Weiterbehandlung der Parkinson-Erkrankung in Anbetracht der psychiatrischen Komorbidität gelang letztlich gestützt durch die gute Bindung des Patienten innerhalb seiner Familie und begleitende sozialpsychiatrische Maßnahmen.

## Fazit

- Bei Patienten mit Parkinson-Erkrankung vor dem 50. Lebensjahr sollte auch bei unauffälliger Familienanamnese bei weiteren klinischen Symptomen an ein Mikrodeletionssyndrom 22q11.2 gedacht werden. Die humangenetische Diagnostik sollte auch die Abklärung von Kopienanzahlvariationen mitbeinhalten.
- Patienten mit 22q11.2-Mikrodeletion sollten auf Parkinson-Symptome kontrolliert werden. Die Behandlung von Psychosen kann die Diagnose erschweren.
- Parkinson-Patienten mit 22q11.2-Mikrodeletion sprechen auf dopaminerge Therapie an, wobei es gehäuft zu Psychosen, Depression und Angstzuständen im Vergleich zum Morbus Parkinson kommt.

## Interessenkonflikt

J. Levin berichtet Referentenhonorare von Bayer Vital, Biogen und Roche, Beratungshonorare von Axon Neuroscience und Biogen, Autorenhonorare von Thieme medical publishers und W. Kohlhammer GmbH medical publishers, nicht-finanzielle Unterstützung von Abbvie und Entschädigung für die Tätigkeit als Teilzeit-CMO von MODAG und als Begünstigter des Phantomaktienprogramms der MODAG GmbH, außerhalb der eingereichten Arbeit. C. Palleis, A. Eißner, S. Förderreuther, K. Bötzel und A. Danek geben an, dass kein Interessenkonflikt besteht.
